# Effects of Heme Modulation on *Ovophis* and *Trimeresurus* Venom Activity in Human Plasma

**DOI:** 10.3390/toxins10080322

**Published:** 2018-08-08

**Authors:** Vance G. Nielsen, Nathaniel Frank, Ryan W. Matika

**Affiliations:** 1 Department of Anesthesiology, University of Arizona College of Medicine, Tucson, AZ 85719, USA; rmatika@anesth.arizona.edu; 2 Mtoxins, 1111 Washington Ave, Oshkosh, WI 54901, USA; nate@mtoxins.com

**Keywords:** hemotoxic venom, thrombelastography, heme, carbon monoxide, metheme

## Abstract

Geographic isolation and other factors result in evolution-driven diversity of the enzymatic composition of venom of pit vipers in the same genus. The present investigation sought to characterize venoms obtained from such genetically diverse *Ovophis* and *Trimeresurus* pit vipers utilizing thrombelastographic coagulation kinetic analyses. The coagulation kinetics of human plasma were assessed after exposure to venom obtained from two *Ovophis* and three *Trimeresurus* species. The potency of each venom was defined (µg/mL required to equivalently change coagulation); additionally, venoms were exposed to carbon monoxide (CO) or a metheme-inducing agent to modulate any enzyme-associated heme. All venoms had fibrinogenolytic activity, with four being CO-inhibitable. While *Ovophis* venoms had similar potency, one demonstrated the presence of a thrombin-like activity, whereas the other demonstrated a thrombin-generating activity. There was a 10-fold difference in potency and 10-fold different vulnerability to CO inhibition between the *Trimeresurus* species. Metheme formation enhanced fibrinogenolytic-like activity in both *Ovophis* species venoms, whereas the three *Trimeresurus* species venoms had fibrinogenolytic-like activity enhanced, inhibited, or not changed. This novel “venom kinetomic” approach has potential to identify clinically relevant enzymatic activity and assess efficacy of antivenoms between genetically and geographically diverse species.

## 1. Introduction

Geographic isolation of venomous snakes within the same genus is associated with evolution-driven diversity of the enzymatic composition of their venom, which is also affected by diet [1,2,3]. A prime example of this sort of diversity are the numerous *Ovophis* and *Trimeresurus* species of Asian pit viper that are scattered across the Indian subcontinent, Southeast Asia, Japanese islands, and islands throughout and just beyond the South China Sea [4]. Based on the diversity of mitochondrial deoxyribonucleic acid, many of these pit vipers, including the ones displayed in Table 1, are posited to be in different genera than they are presently assigned [4]. Proteomic investigation of the effects of geography-driven diversity of venom composition is important, as the toxic enzymes/compounds of any particular venom may or may not be neutralized by antivenom prepared with venom obtained from related but physically separated and genetically distinct species [1,3,4]. Critically, even if the various classes of enzyme (e.g., phospholipase, serine protease, metalloproteinase) within a venom are quantified, proteomic analyses cannot predict which of the enzymes will have the most predominant effect on target molecules (e.g., prothrombin activator, fibrinogenolytic enzyme). Given that coagulopathy following envenomation by snakes with hemotoxic agents continues to be a major cause of international morbidity and mortality [5], identification of the major effects of any specific venom on coagulation provides insight into potential therapies [6,7,8,9,10,11]. Using thrombelastography and human plasma, it has been possible to identify the contribution of diverse venom enzymatic activities on coagulation kinetics between species or within the same venom. Thrombelastographic methods also permit comparisons of the potency of different venoms to achieve the same hemostatic derangement. Further, this method facilitates assessment of the vulnerability of specific venom activities to heme modulation in isolation with carbon monoxide (CO) or *O*-phenylhydroxylamine (PHA), an agent that forms metheme [6,7,8,9,10,11]. Numerous proteins have been found to be heme-bound, and their functions are controlled by the ligand (e.g., oxygen, nitric oxide, CO) that binds to the iron center of the heme group as recently reviewed [12]. Heme modulation has potential therapeutic applications in the setting of envenomation, and we have recently described a heme group bound to a fibrinogenolytic enzyme obtained from *Crotalus atrox* venom, and this enzyme was inhibited by CO [13]. Taken as a whole, the analyses of changes in coagulation kinetics in human plasma mediated by hemotoxic venom can complement the elegant proteomic analyses of these venoms, potentially providing insight into the relative impact of major enzyme types (e.g., snake venom metalloproteinases (SVMP), snake venom serine proteases (SVSP)) on human coagulopathy after envenomation.

Given the preceding, the goals of this study were to: (1) use human plasma to thrombelastographically characterize the effects on coagulation kinetics of hemotoxic venoms obtained from geographically separated and genetically diverse species within the same genus (Table 1); (2) assess any CO-inhibition of venom activities; (3) assess if PHA inhibits venom effects; (4) in the case of prothrombotic venoms, to utilize heparin–antithrombin anticoagulation to separate venom mediated thrombin generation (e.g., prothrombin-activating properties) from thrombin-like activity that is antithrombin resistant. Prior to this investigation, of the species displayed in Table 1, *Ovophis okinavensis* has been noted to have serine proteases and metalloproteinases in its venom, with one enzyme found to be fibrinogenolytic [14,15]; and *Trimeresurus purpureomaculatus* venom was noted to have both thrombin-like and fibrinogenolytic activity [16].

## 2. Results

**Figure 1:**
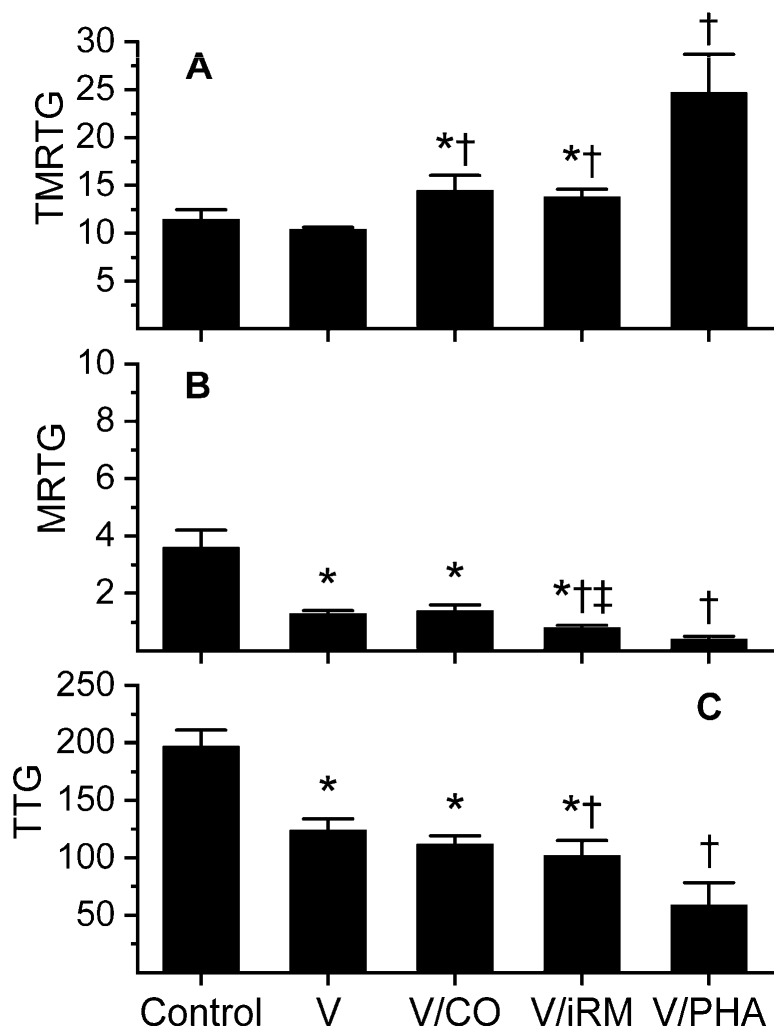
Effects of exposure to carbon monoxide (CO) and O-phenylhydroxylamine (PHA) on *Ovophis monticola* venom activity assessed by changes in coagulation kinetics in human plasma. Data are displayed as mean ± SD. (**A**) time to maximum rate of thrombus generation (TMRTG) units = minutes; (**B**) maximum rate of thrombus generation (MRTG) units = dynes/cm^2^/s; (**C**) total thrombus generation (TTG) units = dynes/cm^2^. Control = plasma without any venom exposure; V = plasma additive-naive venom; V/CO = CO-exposed venom; V/iRM = Venom exposed to iRM (inactivated CORM-2); V/PHA = venom exposed to PHA. * *p* < 0.05 vs. control; † *p* < 0.05 vs. V; ‡ *p* < 0.05 vs. V/CO.

**Figure 2:**
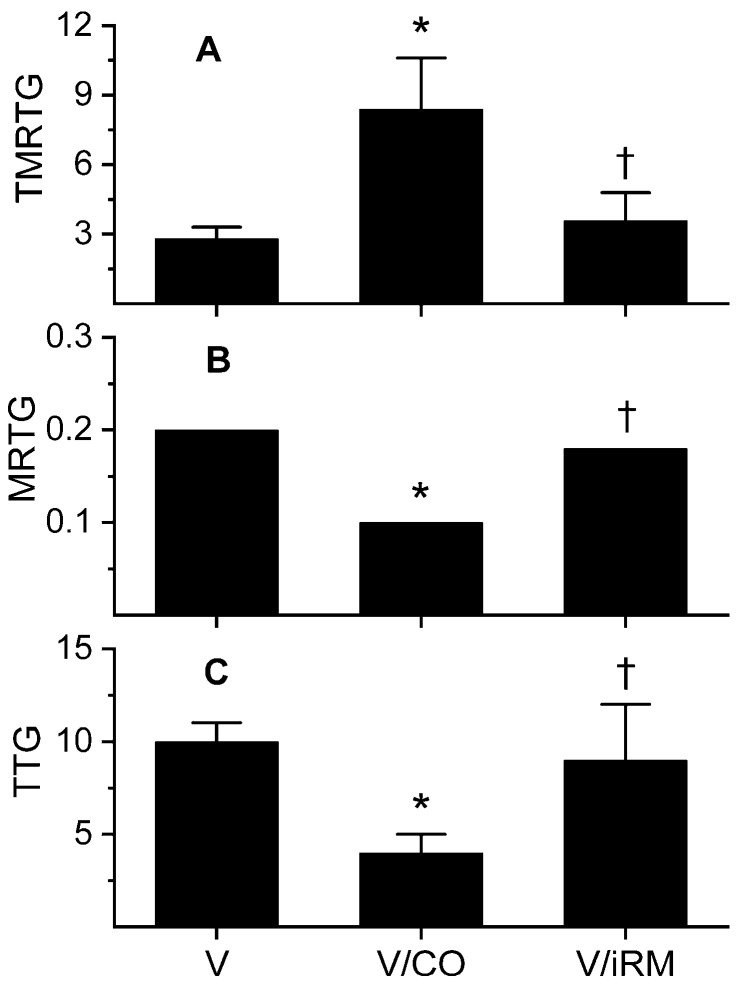
Effects of exposure to CO on *Ovophis monticola* venom activity assessed by changes in coagulation kinetics in human plasma anticoagulated with heparin. Data are presented as mean ± SD. (**A**) TMRTG units = minutes; (**B**) MRTG units = dynes/cm^2^/s; (**C**) TTG units = dynes/cm^2^. V = plasma additive-naive venom; V/CO = CO-exposed venom; V/iRM = Venom exposed to iRM; V/PHA = venom exposed to PHA. * *p* < 0.05 vs. V; † *p* < 0.05 vs. V/CO.

**Figure 3:**
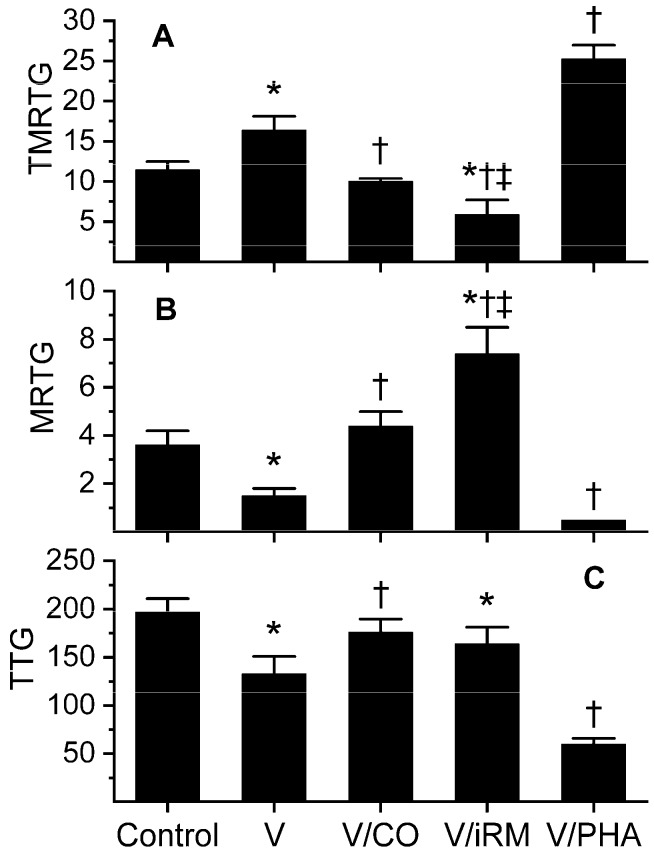
Effects of exposure to CO and PHA on *Ovophis okinavensis* venom activity assessed by changes in coagulation kinetics in human plasma. Data are presented as mean ± SD. **(A)** TMRTG units = minutes; **(B)** MRTG units = dynes/cm^2^/s; **(C)** TTG units = dynes/cm^2^. Control = plasma without any venom exposure; V = plasma additive naive venom; V/CO = CO-exposed venom; V/iRM = Venom exposed to iRM; V/PHA = venom exposed to PHA. * *p* < 0.05 vs. control; † *p* < 0.05 vs. V; ‡ *p* < 0.05 vs. V/CO.

For clarity, the results of each species will be presented in a separate section. Representations of data generated from *Ovophis* species are displayed in Figure 1, Figure 2 and Figure 3, and the results of experiments with *Trimeresurus* species are depicted in Table 2.

### 2.1. Ovophis monticola

Data was generated with a venom concentration of 5 µg/mL in plasma and are displayed in Figure 1. During preliminary work with increasing concentrations of venom, it appeared to be predominantly anticoagulant with decreasing maximum rate of thrombus generation (MRTG, a measure of velocity of clot growth) and total thrombus generation (TTG, a measure of thrombus strength) values; however, time to MRTG (TMRTG, a measure of speed of onset of coagulation) values did not change. Paradoxically, reaction time (R, an early measure of onset of coagulation, minutes) values seemed to decrease with increasing venom concentrations. A venom concentration of 5 µg/mL in the various experimental conditions resulted in R values as follows: control condition, 9.4 ± 0.7 min; venom, 7.0 ± 0.4 min; venom exposed to 100 µM CORM-2 11.0 ± 1.1 min; venom exposed to 100 µM iCORM-2 9.2 ± 0.6 min. All four conditions had R values significantly different from each other, with the exception of control and inactive releasing molecule (iRM)-exposed venom, which were not significantly different. Based on R, this venom had a CO-inhibitable thrombin-like activity in it, with venom accelerating the onset of coagulation and CO prolonging the onset. However, the onset of the CO-exposed venom was more prolonged than control-condition values; thus, there is an opposing anticoagulant enzyme not inhibited by this concentration of CO. Consideration of the remaining parameters in Figure 1 demonstrates this with CO significantly increasing TMRTG values compared to control and venom-alone conditions. However, venom-mediated decreases in MRTG and TTG values were not improved with CO. Of interest, iRM appeared to enhance venom-mediated anticoagulation, evidenced by a greater diminution in MRTG and TTG values compared to venom that was additive naïve. With regard to creating a metheme state with PHA, the anticoagulant effects of the venom were significantly increased compared to additive naïve venom, evidenced by much larger TMRTG values, lesser MRTG, and lesser TTG values. In sum, it appeared that this venom had a CO-inhibitable thrombin-like activity, a CO-resistant but iRM-enhanced fibrinogenolytic-like activity, and a metheme-enhanced fibrinogenolytic-like activity.

Further experiments were performed with plasma-containing heparin, as mentioned previously, with the results displayed in Figure 2. As suspected, an antithrombin-resistant, thrombin-like activity was present that manifested as a rapid in onset, slow-growing, weak thrombus in heparin-anticoagulated plasma. Venom exposure to CORM-2 significantly diminished this thrombin-like activity, but activity of this enzyme was not completely abrogated. Exposure of the venom to iRM resulted in coagulation kinetics not significantly different from samples with additive-naïve venom. In sum, a thrombin-like activity partially antagonized by CO was identified in these experiments. 

### 2.2. Ovophis okinavensis

Data were generated with venom at a concentration of 5 µg/mL in plasma and are depicted in Figure 3. Unlike *O. monticola* venom, this venom demonstrated clear anticoagulant, fibrinogenolytic-like activity, with venom-exposed plasma displaying significantly larger TMRTG values, lesser MRTG values, and lesser TTG values than control-plasma values. These venom-mediated anticoagulant effects were reversed by exposure of the venom to 100 µM CORM-2, resulting in TMRTG, MRTG, and TTG values not different from control-plasma samples. Unexpectedly, exposure of venom to iRM resulted in TMRTG values significantly lesser than the previous three conditions, MRTG values significantly larger than the other conditions, and TTG values significantly lesser than control-plasma samples. This iRM-induced coagulation kinetic profile is consistent with the activation of a thrombin-generating enzyme, similar to that of taipans, as we have previously documented [10]. Lastly, the establishment of a metheme state in the venom resulted in significantly larger TMRTG values, lesser MRTG values and lesser TTG values compared to additive-naïve venom. This pattern was indicative of metheme enhancement of fibrinogenolytic-like activity. Considered as a whole, this venom contained a CO-inhibitable, metheme-enhanced fibrinogenolytic-like activity and an iRM-inducible thrombin-generating activity.

### 2.3. Trimeresurus borneensis

Data were generated with a venom concentration of 1 µg/mL in plasma and are displayed in Table 2. This venom was anticoagulant in nature, with significantly larger TMRTG values, lesser MRTG values, and lesser TTG values in plasma exposed to venom compared to control-plasma sample values. Venom exposed to 100 µM CORM-2 significantly increased MRTG and TTG values compared to additive-naïve venom, and CO-exposed venom lacked TMRTG, MRTG, or TTG values different from control-plasma samples. Exposure of this venom to iRM significantly and markedly enhanced its anticoagulant effect, with a significant elevation in TMRTG, drop in MRTG, and drop in TTG compared to control, venom-alone, or CO-exposed venom conditions. PHA-exposed venom also demonstrated enhanced venom-mediated anticoagulation, with these samples displaying significantly greater TMRTG values, lesser MRTG values, and lesser TTG values than additive-naïve venom-containing samples. In sum, this venom contained a CO-inhibitable fibrinogenolytic activity that was enhanced by iRM and PHA. 

### 2.4. Trimeresurus fasciatus 

Data were generated at a venom concentration of 10 µg/mL in plasma as seen in Table 2. This venom was also anticoagulant in nature, with significantly elevated TMRTG values, dropped MRTG values, and dropped TTG values in plasma exposed to venom compared to control-plasma values. Venom exposed to 100 µM CORM-2 showed no change in activity, so 1 mM CORM-2 was utilized instead. Exposure of the venom to this larger concentration of CORM-2 significantly diminished TMRTG, elevated MRTG values, and elevated TTG values compared to additive-naïve venom, and CO-exposed venom lacked TMRTG, MRTG, or TTG values different from control plasma. Plasma samples with venom exposed to iRM demonstrated TMRTG and MRTG values not different from additive-naïve venom plasma samples; however, TTG values were significantly lesser than the other three conditions. Plasma containing PHA-exposed venom had TMRTG values not different from additive-naïve venom samples; however, plasma with PHA-exposed venom had significantly greater MRTG and TTG values than samples with additive-naïve venom. Considered as a whole, this venom had a CO-inhibitable fibrinogenolytic activity that was PHA-inhibitable. 

### 2.5. Trimeresurus purpureomaculatus

Data were generated at a venom concentration of 5 µg/mL in plasma and are found in Table 2. This venom displayed fibrinogenolytic-like activity, with significantly enlarged TMRTG values, diminished MRTG values, and diminished TTG values in plasma exposed to venom compared to control-plasma values. Venom exposed to 100 µM CORM-2 caused plasma samples to demonstrate TMRTG, MRTG, and TTG values significantly different from plasma containing additive-naïve venom, but not significantly different from control-sample values. Plasma with venom exposed to iRM displayed TMRTG, MRTG, and TTG values not different from additive-naïve venom-containing samples but were significantly different from control and CO-exposed sample values. Further, PHA-exposed venom containing plasma demonstrated no significant differences in TMRTG, MRTG, or TTG values compared to additive-naïve venom-containing plasma. Thus, this venom contained CO-inhibitable, fibrinogenolytic-like activity that was unaffected by either iRM or PHA.

## 3. Discussion

This investigation achieved its stated goals of characterizing the potency, anticoagulant/procoagulant nature, and heme-modulated behavior of these diverse Asian pit viper venoms. Beginning with the physically separated, genetically distinct [4] *Ovophis* species, the two venoms were similar in potency (µg/ml venom to achieve the same coagulation kinetic derangement) with a predominant fibrinogenolytic-like activity exerting the most influence on coagulation. With regard to inhibition by CO, the fibrinogenolytic-like activity of *O. monticola* was resistant to the smaller concentration of CO than that of *O. okinavensis*. The addition of PHA enhanced the fibrinogenolytic-like activity of both species’ venoms. An antithrombin-resistant, CO-inhibitable thrombin-like activity was detectable in *O. monticola* venom, whereas an iRM-enhanced thrombin-generating activity was identified in *O. okinavensis* venom. Critically, the thrombin-generating activity is essentially silent without iRM, with fibrinogenolytic-like activity the major player kinetically in the case of *O. okinavensis* venom. In sum, the venom of the two *Ovophis* species had similar but different characteristics that could be determined thrombelastographically.

With regard to the three genetically distinct [4] *Trimeresurus* species on landmasses separated by the South China Sea, there was a ten-fold difference in potency between the venoms to achieve the same degree of coagulation derangement, which were all fibrinogenolytic-like in nature. However, there were different degrees of susceptibility to CO inhibition of this anticoagulant activity between the venoms. Further, depending on the venom, a metheme state enhanced fibrinogenolytic-like activity, diminished activity, or did nothing to activity. In this genus, iRM remained essentially silent, not interacting with fibrinogenolytic-like activity or enhancing otherwise silent procoagulant activities. Taken as a whole, while the venoms of *Trimeresurus* species primarily had only one predominant anticoagulant activity, the potency and response to heme modulation were remarkably different between vipers.

The use of inactivated CORMs was originally designed to implicate CO-mediated heme modulation, with the concept that the iRM would remain biochemically silent while the CORM would produce an effect in the system tested. Instead, there is a number of works demonstrating CO-independent effects of iRM [7,17,18] wherein various enzymes are modulated via interaction of the iRM without an intermediary attached heme. We have specifically seen northern copperhead fibrinogenolytic-like venom activity enhanced via thrombelastography with iRM [7]. While this may seem confounding, the use of iRM can provide further confirmation of a particular venom activity by enhancing it [7], or as was the case with the present study, decreasing activity (Table 2). Further, as in the case of *O. okinavensis* venom, a previously kinetically silent activity was activated by iRM (Figure 3, a thrombin-generating activity), providing insight into the complexity of this particular venom. In sum, the use of CORMs, iRMs, and other heme modulators may provide direct, heme-mediated insights or heme-independent information as biochemical probes that query the characteristics of any particular venom.

An important issue that was raised in review was the ability of our methodology to discern if the coagulation kinetic profile derived from each venom could provide insight into its enzymatic composition (e.g., SVMP, SVSP). This is unfortunately not possible, as SVMP and SVSP are both capable of fibrinogenolytic, thrombin-like, factor X-activating, prothrombin-activating, and other activities. The relative percentage of any particular enzyme type (e.g., 20% SVMP with 30% SVSP) in any given venom also cannot be determined with our techniques. Thus, the contribution of any particular enzyme by biochemical type is not possible; however, the power of our methods is the revelation as to which of any particular mix of enzymes is the predominant one responsible for the observed coagulopathy in vitro, which, given the milieu (human plasma), would likely predict the clinical situation. When data derived from our methodology are combined with complimentary venomics, the specific enzyme responsible can likely be identified.

The present work focuses on some unique species related by genus in Asia and South East Asia, but in earlier works prior to systematic use of metheme formation [6,7,8,9,10,11], these thrombelastographic methods have been utilized to characterize the coagulation kinetic profiles of medically important species in other parts of the world. North American *Agkistrodon* species venom tested by our laboratory was uniformly anticoagulant in nature, displaying a fibrinogenolytic coagulation kinetic profile [9]. In contrast, while most North American *Crotalus* species venom seemed fibrinogenolytic by our methods [6,7,9], there were some that were procoagulant in nature, displaying thrombin-like activity coagulation kinetic patterns in human plasma [8]. In terms of evolution, the South American *Lachesis muta muta* is considered a progenitor species for pit vipers throughout the Americas, and our methods identified both fibrinogenolytic and thrombin-like activities differentially susceptible to CO inhibition [11]. Of interest, *Naja* species venoms across continents appear to have a highly conserved anticoagulant coagulation kinetic profile that is CO inhibited but may be mediated either by phospholipases and/or SVMP or SVSP [6,10]. Lastly, *Oxyuranus* species in Australia and Papua New Guinea, separated by the Torres Strait, display essentially the same procoagulant, prothrombin activator coagulation kinetic profile that is CO inhibitable [11]. In summary, continued utilization and refinement of our methodology is anticipated to provide insight into the predominant nature and vulnerability to heme modulation of diverse species venoms.

In conclusion, the present work demonstrated that human plasma-based thrombelastographic analyses utilizing heme-modulating approaches and antithrombin activation with heparin can be used to generate a unique coagulation kinetic profile of individual snake venom activities. Just as with the chromatographic and mass-spectroscopic approach with proteomic analyses documenting the presence of key enzymes and compounds to differentiate various venoms [1,3], it may be of utility to similarly kinetically profile the very same venoms to provide a functional assessment of the relative importance of any particular enzyme class (e.g., prothrombin activator, thrombin-like activity, fibrinogenolytic activity) in effecting coagulopathy. These thrombelastograph-based analyses of the impact of various classes of hemotoxic venom activity could perhaps be termed “venom kinetomics”, to complement the specific venomics methods used to assess which particular enzymes of the venom are responsible for coagulopathy. Put another way, while more than one coagulation-modifying activity can be identified in a venom (e.g., fibrinogenolytic-like, thrombin-like activity) with venomics, venom kinetomics can identify which of the enzymes predominate and inflict clinical coagulopathy. This venom-kinetomic approach could also potentially determine the utility of antivenoms across genetically related (same genus) but geographically separated species of venomous snake. Future investigation using this venom kinetomic methodology will demonstrate in time its laboratory and clinical utility.

## 4. Materials and Methods 

### 4.1. Human Plasma, Venoms, and Other Chemicals

Table 1 displays the freeze-dried venoms that were purchased from Mtoxins (Oshkosh, WI, USA). Venoms were dissolved in calcium-free phosphate-buffered saline (PBS, Sigma-Aldrich, Saint Louis, MO, USA) at a final concentration of 50 mg/mL, split into multiple aliquots, and kept at –80 °C until the day of experimentation. CORM-2 (tricarbonyldichlororuthenium (II)dimer, a CO-releasing molecule), dimethyl sulfoxide (DMSO), and PHA were purchased from Sigma-Aldrich. Pooled normal, control human plasma (George King Bio-Medical, Overland Park, KS, USA) anticoagulated with sodium citrate was kept at –80 °C until use in the following studies.

### 4.2. Thrombelastographic Analyses

The volume of all experimental mixtures of chemical additives and plasma was equal to 360 µL. Samples had 320 µL of plasma, 16.4 µL of PBS, 20 µL of 200 mM CaCl_2_, and 3.6 µL of PBS or venom (final concentration species-dependent), which were placed into a disposable cup in a computer-controlled thrombelastograph^®^ hemostasis system (Model 5000, Haemonetics Inc., Braintree, MA, USA) at 37 °C, and then rapidly mixed by moving the cup up against and then away from the plastic pin five times before leaving the mixture between the cup and pin. The following elastic modulus-based parameters previously described [6,7,8,9,10,11] were determined: TMRTG: the time interval (minutes) observed prior to maximum speed of clot growth; MRTG: maximum velocity of clot growth observed (dynes/cm^2^/s); and TTG (dynes/cm^2^), final viscoelastic resistance observed after thrombus growth is complete. For one data set, the R (min), defined as the time to develop 2 mm of amplitude as a measure of the commencement of coagulation, was determined to assess if a venom had a subtle procoagulant component. The period of time for data collection was 20 min with venoms that were procoagulant. Venoms with anticoagulant properties had data collected for 30 min.

### 4.3. Generation of Venom Concentration Experimentation and Effects of Isolated CORM-2 or PHA Venom Exposures

The first concentration for venom assessment was 1 µg/ml. Assuming these venoms to be anticoagulant in nature, the commencement of coagulation had to be at least twice and/or the velocity of clot formation 50% of plasma without venom addition to be satisfactory to further investigate with the venom concentration tested. This is a performance-based model of coagulation kinetic assessment, allowing the ability to compare the concentration of venom required by each species to inflict the same hemostatic derangement. This is the method to estimate the relative potencies of various venoms.

As for exposure to CORM-2 to characterize the effects of CO on venom activity, the subsequent four experimental conditions were utilized: (1) control—no venom, DMSO 1% addition (*v*/*v*) in PBS; (2) venom condition—venom, DMSO 1% addition (*v*/*v*) in PBS; (3) CO-exposed—venom, CORM-2 1% addition in DMSO (100 µM final concentration; and (4) iRM-exposed—venom, inactivated CORM-2 1% addition in DMSO (100 µM final concentration). CORM-2 was inactivated as published elsewhere [19]. If venom was found to not be inhibited by 100 µM CORM-2, a concentration of 1000 µM CORM-2 and iRM was used instead, as this is the greatest concentration utilized to inhibit venom activity prior to in vivo administration [20]. The maximum amount of CORM-2 in the final plasma mixture of these experiments would be 10 µM, which would not be expected to affect coagulation kinetics [21]. Venom was added to PBS with the chemical additions mentioned previously, and after incubation at room temperature for 5 minutes, 3.6 µL of one these four solutions was added to a thrombelastographic cup containing the plasma mixture.

Other experiments involved exposure of all venoms to PHA in order to determine if their activities would vary after metheme (Fe^+3^)-favoring conditions were instituted. This approach has modified the heme state during studies of fibrinogen in vitro [22,23] in human plasma, diminishing the function of fibrinogen as a thrombin substrate. Venom was added to PBS with addition of PHA 3% (*v*/*v*, 30 mM final concentration) for 5 min prior to addition to a thrombelastographic cup containing a plasma mixture. The final concentration of PHA in plasma samples would be 0.3 mM, a concentration not expected to affect plasmatic coagulation [22]. Data generated from this condition were compared to data derived from condition 2 in the immediately preceding experimental series.

### 4.4. Separation of Venom-Mediated Thrombin Generation from Thrombinlike Activity

To isolate the effect on coagulation kinetics of thrombin generated by prothrombin activators or other procoagulant activation dependent on human plasma proteins contained within the venoms from that of direct, thrombin-like activity found in these venoms, additional experiments were performed without or with heparin added to plasma prior to venom exposure. Unfractionated heparin (SAGENT Pharmaceuticals, Schaumburg, IL, USA), (1000 USP U/mL) was placed into plasma samples to achieve a final concentration of 10 U/mL, which is a concentration that will maximally activate antithrombin and inhibit thrombin activity based on previous work in this laboratory [24]. Data were collected for 30 min.

### 4.5. Statistical Analyses

Data are depicted as mean ± SD. A total of *n* = 6 replicates per condition was used in all experimentation, as this results in a statistical power >0.8 with *p* < 0.05 using our thrombelastographic techniques [6,7,8,9,10,11]. A statistical program (SigmaPlot 14, Systat Software, Inc., San Jose, CA, USA) was used for one-way analyses of variance comparisons between conditions, followed by Holm–Sidak post hoc analysis when comparing control, venom, CO, and iRM conditions; comparisons between venom and PHA conditions were performed with unpaired, two-tailed Student’s *t*-tests. Graphics of coagulation kinetic data were generated with graphics software (OrigenPro 2018, OrigenLab Corporation, Northampton, MA, USA; CorelDRAW X8, Corel Corporation, Mountain View, CA, USA). A *p* < 0.05 value was considered statistically significant.

## Figures and Tables

**Table 1:** Diverse Asian pit vipers investigated.

Species	Common Name	Location
*Ovophis monticola*	Mountain Pit Viper	Indian Subcontinent, Southeast Asia
*Ovophis okinavensis*	Okinawa Pit Viper	Ryukyu Islands of Japan
*Trimeresurus borneensis*	Bornean Pit Viper	Brunei, Indonesia, Malaysia, Thailand
*Trimeresurus fasciatus*	Banded Pit Viper	Djampea Island (Indonesia)
*Trimeresurus purpureomaculatus*	Mangrove Pit Viper	Indian Subcontinent, Southeast Asia

**Table 2:** Coagulation kinetics of venoms ± CO or ± PHA in plasma.

	Control	V	V/CO	V/iRM	V/PHA
*Trimeresurus borneensis* (1 µg/mL)					
TMRTG	11.5 ± 1.0	17.8 ± 2.1 *	13.6 ± 3.6	25.2 ± 4.9 * † ‡	23.7 ± 2 †
MRTG	3.6 ± 0.6	1.1 ± 0.3 *	3.2 ± 1.4 †	0.2 ± 0.2 * ‡	0.7 ± 0.1 †
TTG	197 ± 14	144 ± 22 *	186 ± 38 †	28 ± 29 * † ‡	93 ± 19 †
*Trimeresurus fasciatus* (10 µg/mL)					
TMRTG	---	23.2 ± 3.6 *	14.7 ± 3.4 †	24.2 ± 6.5 * ‡	20.0 ± 1.9
MRTG	---	1.0 ± 0.4 *	3.0 ± 0.9 †	0.5 ± 0.6 * ‡	1.8 ± 0.3 †
TTG	---	100 ± 26 *	205 ± 16 †	42 ± 52 * † ‡	166 ± 11 †
*Trimeresurus purpureomaculatus* (5 µg/mL)					
TMRTG	---	22.6 ± 2.2 *	13.9 ± 3.2 †	20.8 ± 1.8 * ‡	21.6 ± 4.1
MRTG	---	0.4 ± 0.2 *	2.7 ± 1.4 †	0.5 ± 0.2 * ‡	0.6 ± 0.2
TTG	---	57 ± 12 *	190 ± 15 †	70 ± 19 * ‡	87 ± 25 †

Data are presented as mean ± SD. Control = no venom or additives; V = venom; V/CO = CO-exposed venom; V/iRM = iRM-exposed venom; V/PHA = PHA-exposed venom. *T. fasciatus* venom was exposed to 1 mM CORM-2/iRM, while the other venoms were exposed to 100 µM CORM-2/iRM. TMRTG units = minutes; MRTG units = dynes/cm^2^/s; TTG units = dynes/cm^2^. * *p* < 0.05 vs. control, † *p* < 0.05 vs. V, ‡ *p* < 0.05 vs. V/CO.
